# The presence of prolines in the flanking region of an immunodominant HIV‐2 gag epitope influences the quality and quantity of the epitope generated

**DOI:** 10.1002/eji.201545451

**Published:** 2015-06-24

**Authors:** Sabelle Jallow, Aleksandra Leligdowicz, Holger B. Kramer, Clayton Onyango, Matthew Cotten, Cynthia Wright, Hilton C. Whittle, Andrew McMichael, Tao Dong, Benedikt M. Kessler, Sarah L. Rowland‐Jones

**Affiliations:** ^1^ Radcliffe Department of MedicineWeatherall Institute of Molecular Medicine, John Radcliffe Hospital, University of OxfordHeadingtonOxfordUK; ^2^ Interdepartmental Division of Critical Care MedicineUniversity of TorontoCanada; ^3^ Department of PhysiologyAnatomy and Genetics, University of OxfordOxfordUK; ^4^ Medical Research Council UnitFajaraThe Gambia; ^5^ Wellcome Trust Sanger InstituteHinxtonCambridgeUK; ^6^ Nuffield Department of MedicineTarget Discovery Institute, University of OxfordOxfordUK; ^7^ Present address: National Research Foundation: Vaccine Preventable Diseases, Respiratory and Meningeal Pathogens Research UnitUniversity of the WitwatersrandSouth Africa

**Keywords:** Antigen processing, CTL epitopes, HIV‐2, Gag p26, DRFYKSLRA

## Abstract

Both the recognition of HIV‐infected cells and the immunogenicity of candidate CTL vaccines depend on the presentation of a peptide epitope at the cell surface, which in turn depends on intracellular antigen processing. Differential antigen processing maybe responsible for the differences in both the quality and the quantity of epitopes produced, influencing the immunodominance hierarchy of viral epitopes. Previously, we showed that the magnitude of the HIV‐2 gag‐specific T‐cell response is inversely correlated with plasma viral load, particularly when responses are directed against an epitope, _165_DRFYKSLRA_173_, within the highly conserved Major Homology Region of gag‐p26. We also showed that the presence of three proline residues, at positions 119, 159 and 178 of gag‐p26, was significantly correlated with low viral load. Since this proline motif was also associated with stronger gag‐specific CTL responses, we investigated the impact of these prolines on proteasomal processing of the protective _165_DRFYKSLRA_173_ epitope. Our data demonstrate that the _165_DRFYKSLRA_173_ epitope is most efficiently processed from precursors that contain two flanking proline residues, found naturally in low viral‐load patients. Superior antigen processing and enhanced presentation may account for the link between infection with HIV‐2 encoding the “PPP‐gag” sequence and both strong gag‐specific CTL responses as well as lower viral load.

## Introduction

Despite approximately 30–60% nucleotide sequence homology between HIV‐1 and HIV‐2, HIV‐2 infection is characterized by slower disease progression, with survival being unaffected by HIV‐2 status for many infected adults 1. Moreover, it was recently shown that preexisting HIV‐2 infection may confer some protection against HIV‐1 disease progression 2. Although the proviral load is similar between HIV‐1‐ and HIV‐2‐infected people at the same stage of infection 3, 4, plasma viral load is significantly lower in most people with HIV‐2 infection 5, 6. However, a minority of HIV‐2‐infected people develop a syndrome characterized by high levels of plasma virus and rapid CD4^+^ T‐cell decline 7, leading to a clinical picture indistinguishable from AIDS caused by HIV‐1 8. Thus HIV‐2 presents the intriguing model of a human retrovirus with which most infected people become long‐term nonprogressors. The discrepancy between the plasma and proviral load in most HIV‐2‐infected patients is compatible with enhanced immune control of viral replication, or defective HIV‐2 replicative capacity, or a combination of both mechanisms. Elucidating the reasons for the relatively attenuated course of HIV‐2 infection, and identifying the key differences between HIV‐2 progressors and nonprogressors, could shed light on the mechanisms of HIV‐1 pathogenesis and provide a better understanding of protective immunity to HIV infection.

Cytotoxic T lymphocytes (CTLs) play a central role in host defense against intracellular pathogens by destroying infected cells presenting peptides processed from the pathogen. CTLs recognize and bind to processed peptides 8–11 residues long that are presented by major histocompatibility complex (MHC) I molecules 9. The role of CTLs in controlling HIV‐1 infection has been well established, with strong supporting evidence 9. The suppressive role of CTLs during HIV‐1 infection is highlighted by viral evasion of immune mechanism via downregulation of host MHC I 10; and mutating within or close to defined CTL epitopes 11, thereby avoiding CTL recognition.

Both the recognition of HIV‐infected cells and the immunogenicity of candidate CTL vaccines depend on the presentation of an epitope peptide at the cell surface 12, which in turn depends on intracellular antigen processing. Differential antigen processing may contribute to both the quantity and quality of epitopes produced 12. Studies in animal models have shown that the efficiency of antigen processing can influence the hierarchy of viral epitopes, suggesting that poorly processed epitopes with a low affinity for the transporter associated with antigen processing (TAP) will be less likely to enter the ER and be loaded onto the MHC 12, 13, 14, and are therefore less likely to be presented to CTLs. In addition, artificial mutations in flanking regions of mouse viral epitopes 15, 16, 17, 18 or naturally occurring mutations in the flanking region of HIV 19, 20 and HCV 21 epitopes have been shown to impair antigen processing and presentation, leading to CTL escape. Le Gall et al. found that a single amino acid change in the flanking sequence of an epitope can either reduce or augment the CTL response significantly 12.

Much less is known about the role of the HIV‐2‐specific CTL response and there have been no reports of the development of HIV‐2 “escape” mutants selected under CTL pressure. Previous studies from our group have shown that HIV‐2‐specific CTL responses have a broader range of functions, particularly at low antigen concentrations, than HIV‐1‐specific T cells 22, 23. Viral control is significantly associated with CD8^+^ T‐cell responses to gag 22: the magnitude of the HIV‐2 gag‐specific T‐cell response is inversely correlated with plasma viral load; particularly when responses are directed against a highly conserved 18 amino acid region of the gag p26 protein, referred to as “peptide 46” 23. Peptide 46 (Gag_298–315_, YVDRFYKSLRAEQTDPAV) is located in the major homology region of the gag protein, a 20‐residue sequence at the C‐terminal domain of the gag capsid 24. This sequence is highly conserved throughout primate lentiviruses and even among most known retroviruses. This evolutionary conservation strongly suggests that the major homology region plays an important role in the retroviral structure and replication 24, 25. The HIV‐1 major homology region contains a number of known MHC class I and class II restricted epitopes. One of the well‐characterized epitopes within peptide 46 is a nonamer, DRFYK**T**LRA 25, 26 in HIV‐1, and DRFYK**S**LRA in HIV‐2, herein referred to as HIV‐1 DA9 (nonamer (9‐mer) epitope within HIV‐2 gag, with the sequence DRFYK**S**LRA) or HIV‐2 DA9, respectively. The HIV‐1 epitope was first reported as the dominant HLA B‐14‐restricted CTL response in a long‐term nonprogressor, infected for over 20 years with HIV‐1[25, 26].

An association between the presence of a motif‐containing 3 prolines (P) residues at positions 119, 159, and 178 of the HIV‐2 capsid (positions 254, 294, and 313, respectively, of HIV‐2 gag gene product, Table 1) and viral control was previously observed in this study population 27. Interestingly, the last two prolines are in the flanking region of the DA9 epitope within peptide 46, [**P**FQSYV**DRFYKSLRA**EQTD**P**AV]. We therefore hypothesized that the presence of prolines in the flanking region of peptide 46 could modulate the quality and quantity of the DA9 epitope generated and therefore affect the magnitude and specificity of the T‐cell response.

**Table 1:** Sequence of peptides containing the immunodominant epitope DRFYKSLRA (DA9)

Peptide name	HIV strain	Position in gag^a^	Position in capsid‐p26/p24 within gag	Sequence (5′‐3′)^b^
P1: HIV‐2‐PP	HIV‐2	285‐318	151‐184	DIKQGPKE**P**FQSYVDRFYKSLRAEQTD**P**AVKNWM
P2: HIV‐2‐SA	HIV‐2	285‐318	151‐184	DIKQGPKE**S**FQSYVDRFYKSLRAEQTD**A**AVKNWM
P3: HIV‐1‐PQ	HIV‐1	284‐317	152‐185	DIRQGPKE**P**F*RD*YVDRFYK*T*LRAEQ*AT* **Q** *E*VKNWM

a Amino acid position with respect to HIV‐1‐HXB2 (acc no. *K03455*) and HIV‐2 ROD (acc no. *M15390*).

b Amino acid in flanking sequence is in bold and shaded, other amino acid substitutions are underlined and in italics.

## Results

### PPP sequence within HIV‐2 capsid is significantly correlated with a higher T‐cell response magnitude

**Figure 1:**
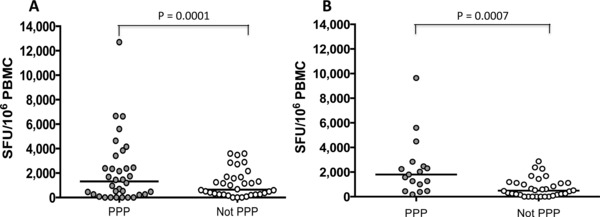
Relationship between IFN‐γ response of HIV‐infected subjects and the presence or absence of prolines at positions 119, 159, and 178 of the HIV‐2 gag (p26) capsid protein. PBMCs from HIV‐infected subjects were stimulated with (A) the entire HIV‐2 proteome or (B) HIV‐2 gag p26 and IFN‐γ production was measured by ELISpot. Each dot represents the response from one individual; 51 samples were assayed once. The average of the spots from the control wells were subtracted from all the sample wells before computing the SFU/10^6^ PBMCs. Black horizontal bars represent median values, and significant *P* values are indicated. Statistical significance calculated using nonparametric Wilcoxon/Mann–Whitney *U* test.

Ex vivo IFN‐γ ELISpot assays were performed using PBMC from HIV‐2‐infected subjects stimulated with 15–19 amino acid long peptides, overlapping by 10 amino acids and spanning a consensus sequence for the HIV‐2 clade A proteome 23. The level of immune response (IFN‐γ production), measured in SFU/10^6^ cells, was compared in patients infected with HIV‐2 whose gag sequences have the PPP motif flanking the peptide 46 region and those with non‐PPP sequences. The same samples were used for both the ELISpot assays and viral sequencing. The results revealed that high magnitude T‐cell responses were significantly correlated with the presence of the PPP motif, for both responses against the entire HIV‐2 proteome (Fig. 1A) and gag‐specific responses (Fig. 1B). All patients were HLA typed and of the 20 subjects tested five (25%) were HLA‐B14 positive. All HLA‐B14 subjects made strong IFN‐γ peptide 46‐specific responses. Three of the five HLA‐B14 subjects had the top highest responses and the remaining two were in the top half of responders (data not shown).

### The CD8^+^ T‐cell epitope within peptide 46 is a nonamer, DRFYKSLRA (DA9)

**Figure 2:**
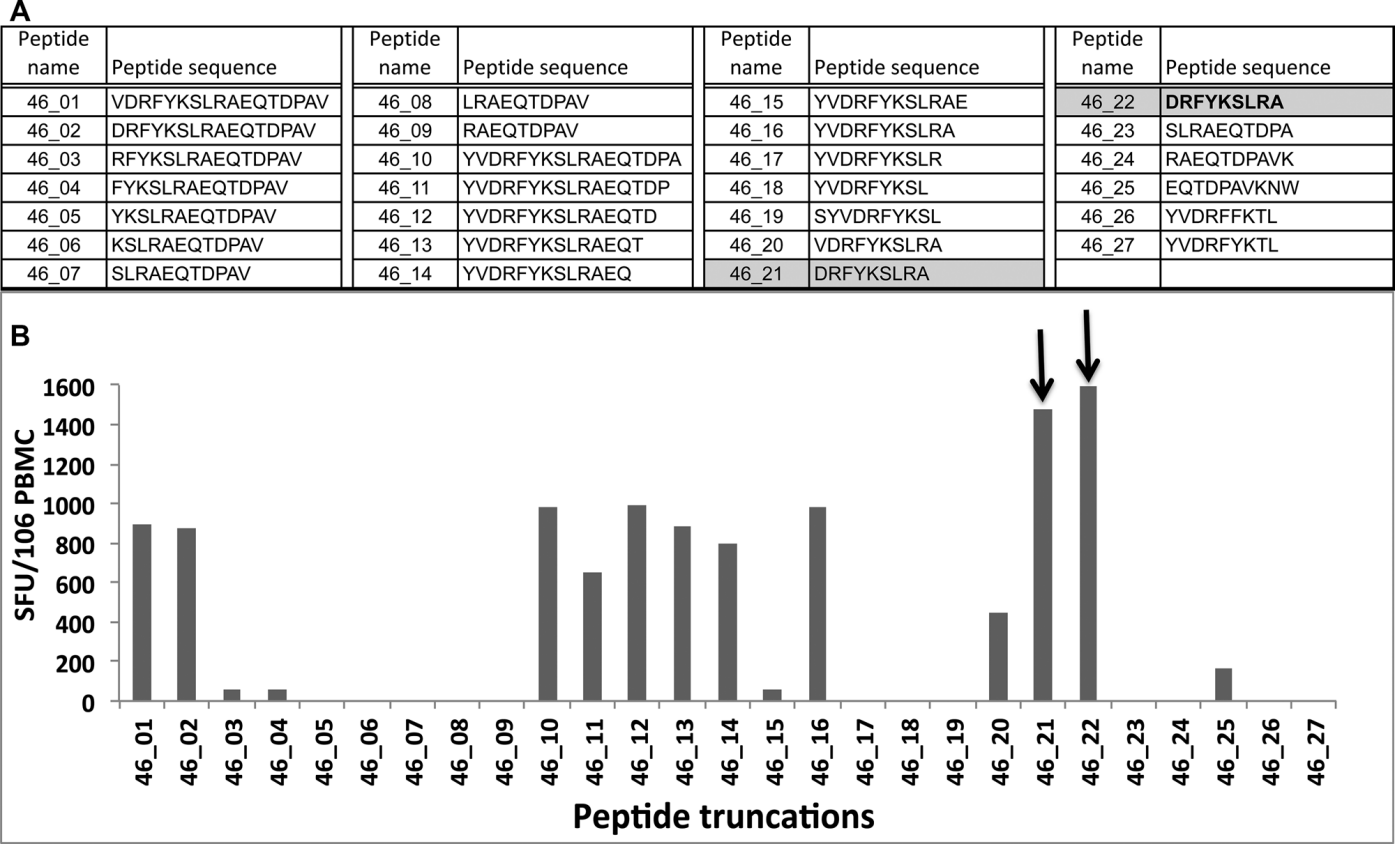
The CD8^+^ T‐cell epitope within peptide 46 is a nonamer, DRFYKSLRA (DA9). Truncations of peptide 46 (YVDRFYKSLRAEQTDPAV) were generated and used in an ex vivo ELISpot together with PBMCs from five HLA‐B14‐positive, peptide 46 responders (CD8^+^ T‐cell response), to determine the epitope length. (A) The sequences of the truncated peptides are shown. The optimal epitope DA9 sequence is highlighted in gray; peptides 46‐21 and 46‐22 have the same sequence, DRFYKSLRA, but were made by different companies. (B) Truncated peptides were used in ex vivo IFN‐γ ELISpot assays to determine the optimal CD8^+^ T‐cell restricted epitope within peptide 46. Each bar represents the number of cells producing IFN‐γ measured in SFU/10^6^ PBMCs. This figure is a representative plot from one individual, assayed once. Four donors were evaluated.

Peptide truncations were generated (Fig. 2A) and used in an ex vivo ELISpot, at a final peptide concentration of 2 μg/mL, together with PBMCs from five HLA‐B14‐positive peptide 46 responders (CD8^+^ T‐cell response), to determine the optimal epitope. Peptides 46‐21 and 46‐22 have the same sequence, DRFYKSLRA, but were made by different companies. The strongest response was observed with the truncated 9‐mer peptide, _165_DRFYKSLRA_173_; suggesting that this peptide represents the optimal epitope (Fig. 2B). In addition, HLA‐B14 tetramer‐sorted CD8**^+^** T‐cell clones, all specific for the DA9 epitope, were successfully obtained from three of the five samples. This result was not surprising, as the equivalent epitope has been previously documented as a CTL epitope in HIV‐1‐infected donors with HLA‐B14 25, 26.

### In vitro generation of DA9 epitope precursor is more efficient for the HIV‐2PP peptide

**Figure 3:**
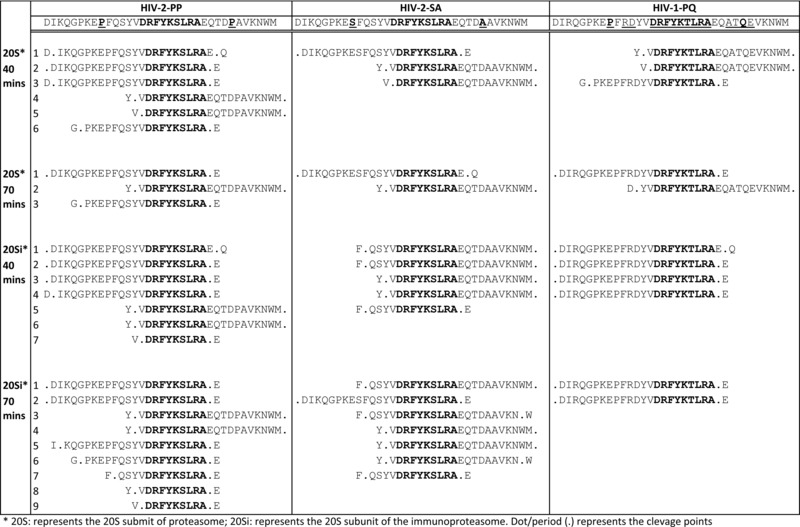
Digestion products of the HIV‐2‐PP, HIV‐2‐SA, and HIV‐1‐PQ peptides analyzed by tandem mass spectrometry. The three 34‐mer peptides—HIV‐2‐PP, HIV‐2‐SA, and HIV‐1‐PQ—were subjected to in vitro digestion with highly purified proteasome and immunoproteasome for (A) 40 min and (B) 70 min. Proteolysis products were evaluated by tandem mass spectrometry to measure production efficiency. The proteolytic products containing intact DA9 sequences, called epitope precursors, are shown for each digestion experiment. The amino acid substitutions in the HIV‐2‐PP, HIV‐2‐SA, and HIV‐1‐PQ peptides are in bold and underlined, other differences in sequence are underlined, and DA9 in each epitope precursor is bold. The dots around the start and end of the sequences represent the N‐ and C‐ terminal cleavage sites, respectively.

**Figure 4:**
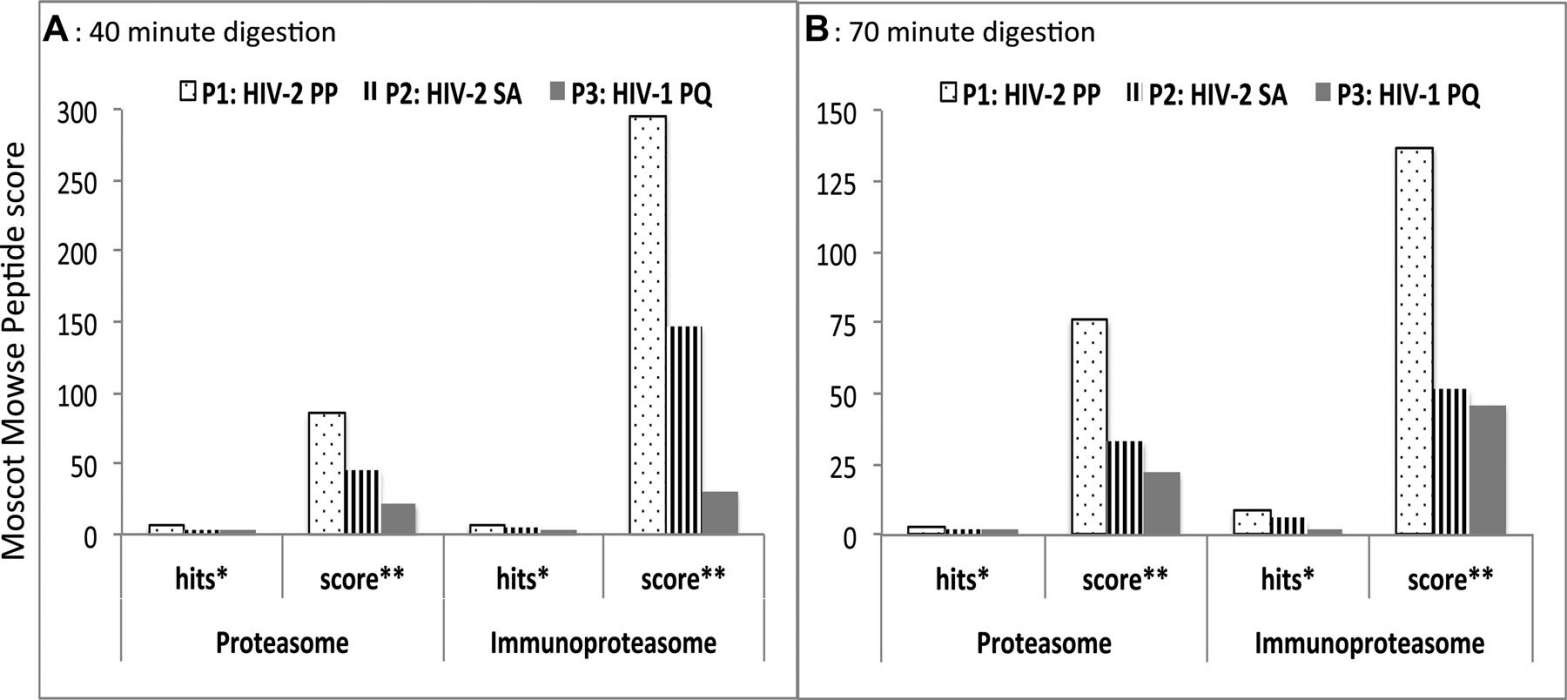
In vitro generation of DA9 epitope precursor is more efficient for the HIV‐2PP peptide. The three 34‐mer peptides—HIV‐2‐PP, HIV‐2‐SA, and HIV‐1‐PQ—were subjected to in vitro digestion with highly purified proteasome and immunoproteasome for (A) 40 min and (B) 70 min. Proteolysis products were evaluated by tandem mass spectrometry to measure production efficiency. P1: HIV‐2‐PP represents 34‐mer HIV‐2 peptide containing prolines at positions 159 and 178; P2: HIV‐2‐SA represents another 34‐mer HIV‐2 peptide of the same sequence except it is flanked by serine (S) and alanine (A) at positions 159 and 178; and P3: HIV‐1‐PQ is an equivalent HIV‐1 34‐mer peptide from the HXB2 sequence (accession no: K03455). Semiquantitative information for the relative abundance of peptide species observed between different experimental conditions was obtained by comparing Mascot peptide Mowse scores. Hits (*) or epitope precursors represent the “number” of peptides <25 amino acids long that contain the intact DA9 epitope (Fig. 3). Score (**): represents the “sum” of peptide identification confidence (Mascot Mowes) scores of peptides <25 amino acids long containing the DA9 epitope. Data are presented as Mascot peptide Mowse scores and data shown are from a single experiment.

To evaluate the effect of the PP motif present in the flanking regions of the DA9 epitope on antigen processing, we synthesized three 34‐mer peptides containing the DA9 epitope in the middle. To generate our peptides, we aligned HIV‐2 p26 sequences from Caio, Guinea Bissau, accession numbers: GQ485448‐GQ485516 27, as well as other HIV‐2 p26 sequences from different HIV‐2 groups available online. Using these alignments, we designed two representative HIV‐2 peptides (Table 1), differing by only two amino acids at positions 159 and 178. HIV‐2‐PP represents a 34‐mer HIV‐2 peptide containing prolines at positions 159 and 178; and HIV‐2‐SA represents another 34‐mer HIV‐2 peptide of the same sequence except it is flanked by serine (S) and alanine (A) at positions 159 and 178 (S and A are the most common amino acids, apart from prolines, at positions 159 and 178; see Materials and methods). HIV‐1‐PQ is an equivalent HIV‐1 peptide from the HXB2 (accession no: K03455) sequence. We subjected these peptides to in vitro digestion with highly purified proteasome (isolated from human erythrocytes) and immunoproteasome (isolated from human spleen) and analyzed the proteolysis products by tandem mass spectrometry. Semiquantitative information for the relative abundance of peptide species observed between different experimental conditions was obtained by comparing Mascot peptide Mowse scores. We computed the number of peptide cleavage products that were at most 24 amino acids long and still contained the DA9 epitope (epitope precursors); and reported them as Hits (the number of epitope precursors with less than 25 amino acids long that contain the intact DA9 epitope); and Scores (the sum of peptide identification confidence (Mascot Mowes) scores of peptides less than 25 amino acids long containing the DA9 epitope). After 40 min of digestion with proteasome, we observed six, three, and three epitope precursors for the HIV‐2‐PP, HIV‐2‐SA, and HIV‐1‐PQ peptides, respectively (Fig. 4A). The 70‐min proteasomal digestion yielded three, two, and two epitope precursors for the HIV‐2‐PP, HIV‐2‐SA, and HIV‐1‐PQ peptides, respectively (Figs. 3 and 4B). At both time points, the HIV‐2‐PP peptide yielded the epitope precursors with the highest scores, followed by HIV‐2SA and HIV‐1PQ (Figs. 3 and 4). Digestion with the immunoproteasome was generally more efficient (Figs. 3 and 4) in our experiment, yielding seven, five, and four epitope precursors after 40 min and nine, seven, two precursors after 70 min for the HIV‐2‐PP, HIV‐2‐SA, and HIV‐1‐PQ peptide precursors, respectively. Following the same trend, the HIV‐2‐PP peptide digested with the immunoproteasome generated epitope precursors with the highest scores, which were much higher than those observed with the corresponding proteasome digestions (Fig. 4).

Taken together, both the proteasomal and immunoproteasomal digestions produced more N‐ and C‐terminally extended DA9 precursors derived from the HIV‐2‐PP peptide as compared to the HIV‐2‐SA peptide. Also, both HIV‐2 peptides produced more precursors than the equivalent HIV‐1 peptide (Fig. 3). These epitope precursors will further undergo N‐terminal cleavage and exact C‐terminal cleavage before being transported to the ER for further processing and loading onto MHC I molecules for antigen presentation on the cell surface.

### Exact C‐terminal cleavage was most efficient for the HIV‐2 peptide with the PP motif

**Figure 5:**
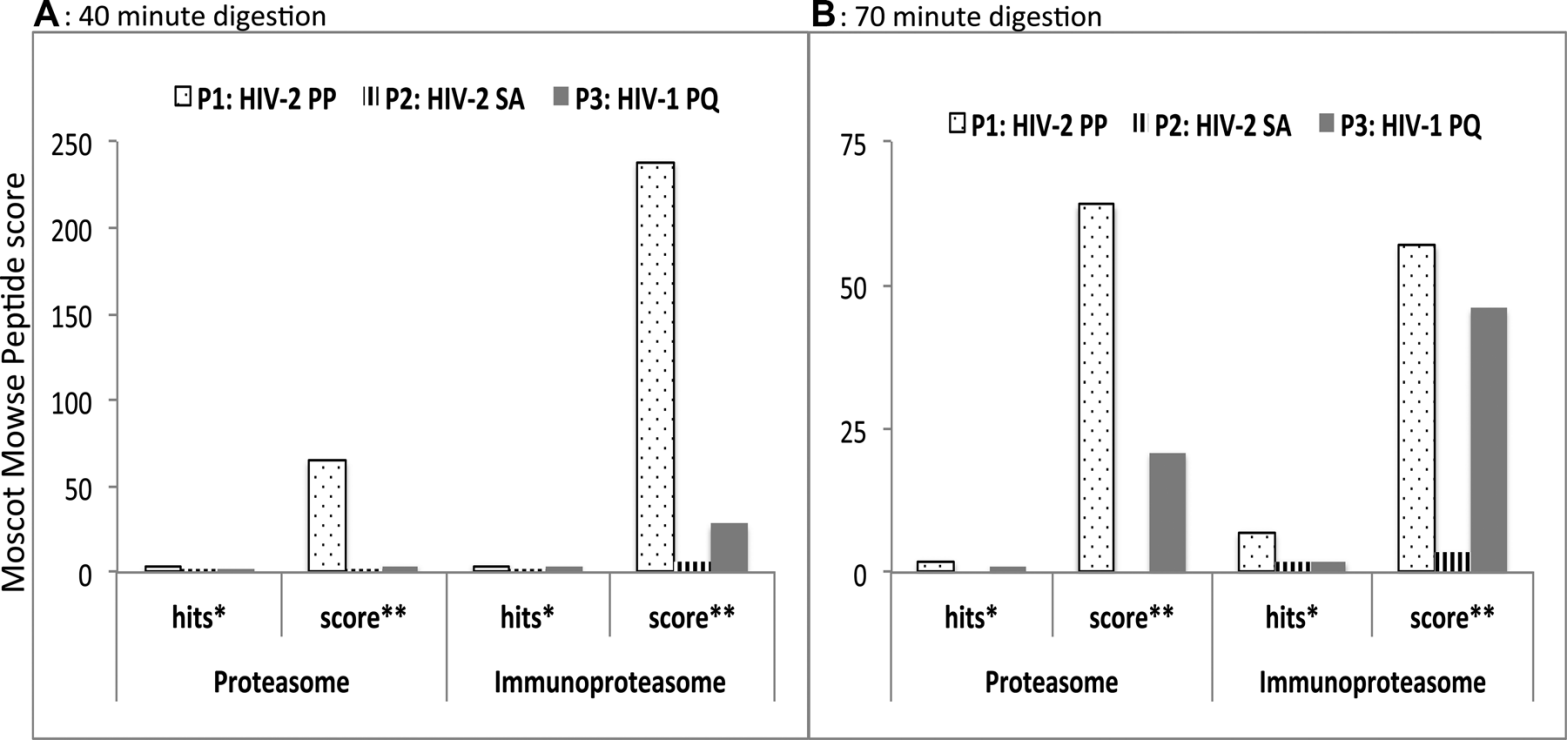
Exact C‐terminal cleavage was most efficient for the HIV‐2 peptide with the PP motif. The three 34‐mer peptides—HIV‐2‐PP, HIV‐2‐SA, and HIV‐1‐PQ—were subjected to in vitro digestion with highly purified proteasome and immunoproteasome for (A) 40 min and (B) 70 min. Proteolysis products were evaluated by tandem mass spectrometry to measure efficient C‐terminal cleavage. P1: HIV‐2‐PP, P2: HIV‐2‐SA, and P3: HIV‐1‐PQ are the 34‐mer peptide sequences as described in the legend of Figure 4 or Table 1. Hits (*) and Score (**) are defined as for Figure 4. Data are presented as Mascot peptide Mowse scores and data shown are from a single experiment.

Proteasomes effectively and precisely cleave CTL epitope peptides at the C‐terminal end 28 and may leave an N‐terminal extension that can be further trimmed by aminopeptidases in the cytoplasm or endoplasmic reticulum (ER) to generate peptides 8–11 amino acids in length 29. In addition, a large cytosolic peptidase, tripeptidyl peptidase II (TPPII) removes groups of three residues from the N‐terminus of peptides at least 16 amino acids long 30. Although C‐ and N‐terminally extended peptides may be generated and transported into the ER, only those with N‐terminal extensions are presented by MHC I 31. Thus, we analyzed the fragments for exact C‐terminal cleavage. Mass spectrometry analysis revealed that for both the proteasomal and immunoproteasomal digestions, the HIV‐2‐PP peptide was cleaved most efficiently at the C‐terminus, followed by HIV‐1‐PQ, and cleavage was least efficient for HIV‐2‐SA, and this was observed at all time points (Fig. 5).

### The exact nonamer epitope (DA9) was produced only from the HIV‐2 peptide with the PP motif

**Figure 6:**
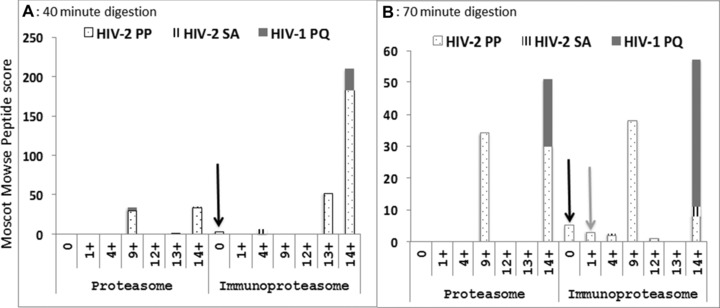
The exact nonamer epitope (DA9) is produced only from the HIV‐2 peptide with the PP motif. The three 34‐mer peptides generated from epitope DA9—HIV‐2‐PP, HIV‐2‐SA, and HIV‐1‐PQ—were subjected to in vitro digestion with highly purified proteasome and immunoproteasome for (A) 40 min and (B) 70 min. Proteolysis products were evaluated by tandem mass spectrometry to measure the quantity of peptides produced with exact C‐terminal cleavage, and with N‐terminal extensions of up to 14 residues. P1: HIV‐2‐PP, P2: HIV‐2‐SA, and P3: HIV‐1‐PQ are the 34‐mer peptide sequences as described in the legend of Figure 4 or Table 1. Hits (*) and Score (**) are defined as for Figure 4. Data are presented as Mascot peptide Mowse scores and data shown are from a single experiment.

Previous research has indicated that some MHC I molecules can directly bind peptides generated by either the proteasomes or immunoproteasome and transport them to the ER using TAP 32, while other extended peptides are further trimmed in the ER or cytosol. Those further trimmed in the cytosol have to reenter the peptide‐loading complex and compete with newly translocated peptides 32. Since exact C‐terminal cleavage is necessary for presentation, we further analyzed the N‐terminal cleavages of peptides with a perfect C‐terminal cleavage, by considering up to 14 extra residues extending from the N‐terminal of the DA9 epitope. N‐terminal processing was also most efficient with the HIV‐2‐PP peptide with exact or short N‐terminal extensions, followed by HIV‐1‐PQ and, again, worst for HIV‐2‐SA (Figs. 3 and 6). Analysis of the peptides showed that the generation of the exact 9‐mer, DA9 (N‐terminal extension = 0), was indeed only present in digests from the HIV‐2‐PP peptide, with the immunoproteasome after both the 40‐ and 70‐min digestions (Fig. 6). In addition, a 10‐mer (1+ DA9) N‐terminal extended peptide was found after 70 min digestion of the HIV‐2‐PP peptide with the immunoproteasome (Figs. 3 and 6)

### The immunoproteasome is much more efficient in generating the DA9 epitope in vitro

While the proteasome is adept at processing peptides for class I presentation, the immunoproteasome is much more efficient at this process 33. Immunoproteasome cleavage is characterized by reduced cleavage after acidic residues and increased cleavage after basic and large hydrophobic residues, which are favored by TAP and found in peptides efficiently bound to MHC I molecules 33. In our experiments, we employed both types because it has been observed that, in some instances, it is the proteasome and not the immunoproteasome that generates certain epitopes 33. We found that both the proteasome and immunoproteasome generated the N‐ and C‐terminal extended epitope precursors (Fig. 4), but the immunoproteasome produced much higher relative amounts of the epitope precursors and was able to produce the exact C‐ and N‐terminal cleavages including the exact 9‐mer epitope (Figs. 3 and 6).

## Discussion

This study shows that the presence of prolines in the flanking region of peptide 46 influences the quality and quantity of the DA9 epitope generated. The HIV‐2‐PP and HIV‐2‐SA peptides differ by only two amino acids (**P159S** and **P178A**), yet both proteasomal and immunoproteasomal digestions produce the DA9 epitope more efficiently for HIV‐2‐PP, which has two flanking prolines. The superior antigen processing of HIV‐2‐PP relative to HIV‐2‐SA is shown by the higher abundance of DA9 epitope precursors, exact C‐terminal cleavage, better N‐terminal cleavage, and generation of the optimal 9‐mer epitope. These results indicate that the significant correlation previously found between the presence of prolines at positions 119, 159, and 178 in the HIV‐2 capsid and low viral load 27, as well as the strong gag‐specific responses in patients infected with HIV‐2 viruses with the PPP capsid both correlate with superior antigen processing in vitro of the DA9 epitope flanked by two proline residues.

The role of CTLs in the control of HIV‐1 replication is well established and has led to numerous efforts to generate candidate HIV vaccines that would elicit potent CTL responses. However, due to the difficulties of using whole virus, most studies use peptide pulsing to detect or quantify CTL responses. Although useful for screening studies, this approach overlooks a few critical steps in the antigen‐processing pathway 12. A limitation to our study is that in vivo, peptides generated by the proteasome or immunoproteasome may be later subjected to further degradation by cytosolic peptidases, and hence it cannot be excluded that the DA9 epitope within the extended peptides or even the actual epitope, observed in our studies, may be destroyed before it is presented. However, due to the central role the proteasome plays in epitope generation, and the fact that the proteasome has been known to generate correctly cleaved epitopes that are presented 33, 34, 35, indicates that in vitro proteasomal digestions can be reliably used 36, 37, 38. Upon discovery of a significant correlation between responses to peptide 46 and HIV‐2 viral control, we tried to decipher why only some individuals responded to this epitope and not others, despite a high degree of epitope conservation. The observation that the presence of prolines flanking this HLA‐B14 epitope was also linked to HIV‐2 control indicated that differential antigen processing due to prolines in the flanking region may explain the response, or lack thereof, to peptide 46, irrespective of epitope conservation. We performed in vitro proteasomal degradation of long synthetic peptides and found that antigen processing plays an important role in the generation of the HIV‐2 DA9 epitope. The peptide with two flanking prolines, HIV‐2‐PP, was processed much more efficiently than both HIV‐2‐SA and HIV‐1‐PQ. Even though for the HIV‐2‐SA peptide, we observed more epitope precursors than for the HIV‐1 peptide, HIV‐1‐PQ, processing was more efficient for HIV‐1‐PQ because both the C‐ and N‐ terminal cleavages responsible for DA9 epitope generation occurred more efficiently for HIV‐1‐PQ. Thus we conclude that both the previously observed correlations between the presence of three prolines in the HIV‐2 capsid and low viral load, and the greater magnitude T‐cell responses in individuals infected with HIV‐2 strains that have these prolines in the region flanking the DA9 epitope (HIV‐2‐PP), reflect the superior antigen processing of the HIV‐2‐PPP gag protein by the immunoproteasome.

Proline is unique among the other 19 amino acids in that it is the only cyclic amino acid (its amine nitrogen is not bound to a hydrogen atom within peptide sequences). Furthermore proline residues can adopt cis/trans‐peptide bonds, requiring cells to utilize prolyl isomerases to facilitate protein folding 39. These features and its cyclic nature mean that proline is unique structurally in a way that makes it important in protein folding. The cyclic structure locks its Φ backbone making it conformationally rigid, such that when found within secondary structures such as alpha helices and beta sheets, it forms a kink in the helix or sheet, disrupting their stability and structure 39, 40. The prolines flanking the DA9 epitope are located in the C′‐end of the capsid, involved in dimer formation and capsid shell assembly 41. Onyango et al. modeled and generated p26 capsid proteins including various combinations of proline residues observed in the HIV‐2 isolates from Caio; and upon comparing the binding energies of the different capsids, found that PPP capsids had statistically weaker binding energies compared to those without prolines 27. The lower binding energy of the PPP capsid suggests lower dimer stability that may also have consequences for antigen processing, and this mechanism may be additive to the direct influence on proteosomal processing that was documented in the current study 23. Unstable proteins are probably more susceptible to proteasomal degradation, leading to better processing and therefore presentation of epitopes from such proteins 42 and hence the presence of prolines flanking an epitope may increase epitope production via this mechanism. In linear sequences, the presence of proline at a cleavage site (P1) usually inhibits proteolysis, as most proteases do not accept proline at this site 43, 44. However, the presence of proline at P2 or P4 can sometimes promote proteolysis at the cleavage site (P1‐P1′) 44, 45.

Several HIV vaccines are in various stages of development. These include CTL vaccines made up of strings of conserved HIV dominant or subdominant epitopes in an immunogenic vector. Our study shows that sequence conservation of an epitope is not sufficient for it to be immunogenic; and that the flanking sequences also need to be taken into account as they may profoundly influence efficient processing and presentation. It may be the case that the presence of prolines flanking certain epitopes can result in better antigen processing; this could be further explored in the generation of HIV epitope vaccines. Future studies focusing on the elucidation of the mechanisms of viral control in HIV‐2 infection, as well as a better understanding of the factors governing epitope processing and presentation, will be useful in vaccine design and in the development of new therapeutics against HIV infection.

## Materials and methods

### Study population

The Caió cohort in rural Guinea Bissau was initiated in 1988 to study the epidemiology of HIV‐2 infection. Since then, several serosurveys of the entire community have been performed to record incident HIV‐1/2 infection. Between each serosurvey, HIV‐infected individuals were followed up more regularly and had free access to clinical care. All cohort members were antiretroviral therapy naive until the introduction of antiretroviral therapy in 2007 as part of the national program in Guinea‐Bissau. HIV‐2 infected individuals were sampled from this cohort as previously described 23, 27. Study participants provided consent; the joint Gambian Government Medical Research Council Ethics Committee and the National AIDS Control Programme Committee of Guinea Bissau approved the studies.

### Ex‐vivo IFN‐γ ELISpot assays

A total of 96‐well MultiScreen filter plates (Millipore) were coated with 15 μg/mL of anti‐IFN‐γ monoclonal antibody (1‐DIK, Mabtech). A total of 10^5^ PBMCs/well in 80 μL of H10 medium (RPMI 1640, 10% human AB serum, 2 mM L‐glutamine, 50 U/mL penicillin/streptomycin [Sigma Aldrich]) were added to the plates. Cells were then stimulated with 20 μL of 10 μg/mL of peptide for 16 h as previously described 23. Spots on the plates were counted using an AID ELISpot reader system (Autoimmun Diagnostika GmbH). Results were expressed as spot forming units (SFU)/10^6^ PBMCs. Each dot represents the response from one individual; 51 samples were assayed once. The average of the spots from the control wells were subtracted from all the sample wells before computing SFU/10^6^ PBMCs. Results were regarded as positive if they were at least three times the mean of the quadruplicate negative control wells and over 50 SFU/10^6^ PBMCs. If background wells were more than 30 SFU/10^6^ PBMCs or if both positive control wells (phytohemagglutinin or FEC stimulation) were negative, the assay was excluded from further analysis. Peptides based on truncations of peptide 46 were generated (Fig. 2A) and used in an ex vivo ELISpot assay, at a final peptide concentration of 2 μg/mL, together with PBMCs from five HLA‐B14 positive peptide 46 responders (CD8**^+^** T‐cell response). Peptides 46‐21 and 46‐22 have the same sequence, DRFYKSLRA, but were made by different companies.

### Peptide design

By stratifying capsid sequences according to the presence or absence of prolines at positions 119, 159, and 178 of the HIV‐2 capsid, Onyango et al. found a significant correlation with low plasma viral load 27. At position 119, the other amino acids present were **alanine** (*n* = 38), glutamine (*n* = 5), or glycine (*n* = 1); at position 159 the nonproline amino acids were **serine** (*n* = 29) and threonine (*n* = 2); and at the last position, 178, there were **alanine** (*n* = 18), serine (*n* = 2), glutamine (*n* = 1), and valine (*n* = 1). To generate our peptides, we aligned HIV‐2 p26 sequences from Caio, Guinea Bissau, accession numbers: GQ485448–GQ485516 27, first according to response to peptide 46, and then in order of increasing viral load in each group (responders and nonresponders). We also included HIV‐2 p26 sequences from different HIV‐2 groups available online. Using these alignments, we designed two representative HIV‐2 peptides (Table 1), each 34 amino acids long, but differing by only two amino acids at positions 159 and 178, respectively, one with two prolines_159/178_ (PP) and the other with serine_159_ and alanine_178_ (S and A are the most common amino acids at positions 159 and 178). HIV‐1‐PQ is an equivalent HIV‐1 34‐mer peptide from the HXB2 sequence (accession no: K03455). The long peptides were each produced to >99% purity (Biomatik, Ontario, Canada). Individual truncated peptides used in ELISpot assays were synthesized in‐house by 9‐fluorenylmethoxy carbonyl (Fmoc) chemistry, at the Weatherall Institute of Molecular Medicine, the University of Oxford and the peptide sequences confirmed by MALDI‐TOF/TOF mass spectrometry analysis.

### In vitro proteasomal digestion

Highly purified 20S proteasome (cat no: BML‐PW8720‐0050), isolated from human erythrocytes and 20S immunoproteasome (BML‐PW9645‐0050) isolated from human spleen, bought from Biomol International, Enzo Life sciences, were used in our experiments. The certificates of analysis included Western blots to show the purity of the proteasomes and immunoproteasomes. One microgram of either purified human or was added to 10 μg of each 34‐mer oligopeptide in a final volume of 100 μL of proteolysis buffer (20 mM HEPES (pH 7.8), 2 mM magnesium acetate and 2 mM dithiothreitol) and incubated at 37°C in 5% CO_2_ for 0, 40, and 70 min, after which the reaction was stopped by adding 10 μL formic acid. The samples were kept frozen at −80°C until analysis 36. To eliminate nonspecific degradation, we included two sets of controls in each experiment: one with peptide only, without enzymes (proteasome or immunoproteasome) in the proteolytic buffer and the other with enzymes included, but with formic acid added immediately to stop the enzymatic reaction. Digested samples were desalted using C18 Zip‐tip (Millipore) according to the manufacturer's instructions and concentrated by vacuum centrifugation. Samples were then analyzed by nanoliquid chromatography mass spectrometry using a nano‐Acquity UPLC coupled to a Waters quadruple time‐of‐flight (Q‐Tof) Premier tandem mass spectrometer (Waters, Milford, MA, USA) as described previously 46. To determine peptide precursors, fragment identification and peptide quantification, the instrument was configured to analyze data in high/low collision switching (MS^E^) mode 46. ProteinLynx Global Server Software (v.2.3) was used to convert MS^E^ data into MS/MS spectra. The MS/MS spectra (peaklists) were searched against modified databases containing SwissProt database (release version 54.0, 07/2007, number of entries 276 256) with additional custom HIV‐1 and HIV‐2 sequences using Mascot version 2.2 (Matrix science, London, UK). In addition, individual MS/MS spectra for peptides with a Mascot Mowse score lower than 40 (Expect < 0.015) were inspected manually and included in the quantification only if a series of at least four continuous *y* or *b*‐ions were observed, according to published guidelines 47, 48. The local “in‐house” Mascot server used for this study is supported and maintained by the Computational Biology Research Group at the University of Oxford. Semiquantitative information for the relative abundance of peptide species observed between different experimental conditions was obtained by comparing Mascot peptide Mowse scores 48. The controls, no enzyme added or digestion stopped immediately, were used to eliminate potential degradation products.

## Conflict of interest

The authors declare no financial or commercial conflict of interest.

**Abbreviations**
DA9: nonamer (9‐mer) epitope within HIV‐2 gag, with the sequence DRFYK**S**LRA

## Supporting information

As a service to our authors and readers, this journal provides supporting information supplied by the authors. Such materials are peer reviewed and may be re‐organized for online delivery, but are not copy‐edited or typeset. Technical support issues arising from supporting information (other than missing files) should be addressed to the authors.

Immunoproteasome 20S (human), (purified)

Peer review correspondence
