# Correction

**DOI:** 10.1002/eji.201570096

**Published:** 2015-09-07

**Vol 45 (08) 2015** | DOI: 10.1002/eji.201545451

**The presence of prolines in the flanking region of an immunodominant HIV‐2 gag epitope influences the quality and quantity of the epitope generated**

Sabelle Jallow, Aleksandra Leligdowicz, Holger B. Kramer, Clayton Onyango, Matthew Cotten, Cynthia Wright, Hilton C. Whittle, Andrew McMichael, Tao Dong, Benedikt M. Kessler and Sarah L. Rowland‐Jones

The copyright line of the above article has been changed since first published on 24 June 2015 from the standard copyright to CC‐BY.

The correct copyright of this article is:

© 2015 The Authors. *European Journal of Immunology* published by Wiley‐VCH Verlag GmbH & Co. KGaA, Weinheim.

This is an open access article under the terms of the Creative Commons Attribution License, which permits use, distribution and reproduction in any medium, provided the original work is properly cited.

