# Supplementary material for: The presence of prolines in the flanking region of an immunodominant HIV‐2 gag epitope influences the quality and quantity of the epitope generated
Source: Eur J Immunol. 2015 Jun 24;45(8):2232–42. doi: 10.1002/eji.201545451 (PMC4832300; doi:10.1002/eji.201545451)
Supplement: Supplementary file 1 — Immunoproteasome 20S (human), (purified) [file EJI-45-2232-s001.pdf]

PRODUCT DATA SHEET

Immunoproteasome 20S (human), (purified)

BML-PW9645

Product Number/Sizes

BML-PW9645-0050	50 µg

Product Specifications

SOURCE:	Isolated from human spleen.
FORMULATION:	Liquid. In TEAD buffer (20mM TRIS/HCl, 1mM sodium azide, 1mM DTT, pH 7.4).
PURITY DETAIL:	Highly purified. All starting material has been tested and found to be negative for hepatitis B surface antigen, human immunodeficiency virus type 1 antigens, and antibodies against human immunodeficiency viruses type 1 and 2, and hepatitis C virus.
LONG TERM STORAGE:	-80°C
USE/STABILITY:	Once thawed the material can be stored at 4°C for up to 3 months, or for longer term at -20°C with the addition of up to 50% glycerol to prevent freezing.
HANDLING:	Avoid freeze/thaw cycles.
MISCELLANEOUS/GENERAL:	The eukaryotic proteasome contains 7α-type and 7 β-type subunits. Upon stimulation with IFN-γ, the three active β subunits, β1 (Y), β2 (Z) and β5 (X) are exchanged to their immunocounterparts, namely β1i (LMP-2), β2i (MECL-1) and β5i (LMP-7). This results in a change in proteasomal substrate specificity. Some groups have reported increases in chymotrypsin-like and trypsin-like activities while others have reported no changes or even decreased activities after such treatment.

GLOBAL HEADQUARTERS

Enzo Life Sciences, Inc.
10 Executive Blvd
Farmingdale, NY 11735
USA
T 1-800-942-0430
T 1-160-941-0430
F 1-610-941-9252
E info-usa@enzolifesciences.com
www.enzolifesciences.com

EUROPE/ASIA

Enzo Life Sciences (ELS) AG
Industriestrasse 17, Postfach
CH-4415 Lausen
Switzerland
T +41/061 926 89 89
F +41/061 926 89 79
E info-ch@enzolifesciences.com
www.enzolifesciences.com

For Research Use Only, Not for Human

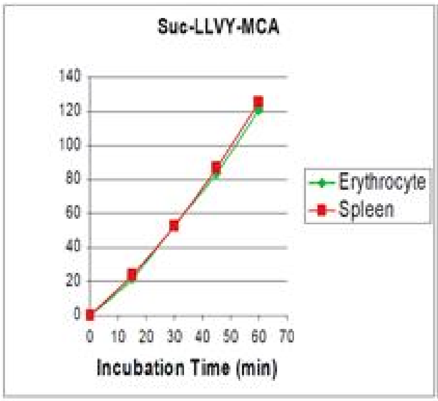

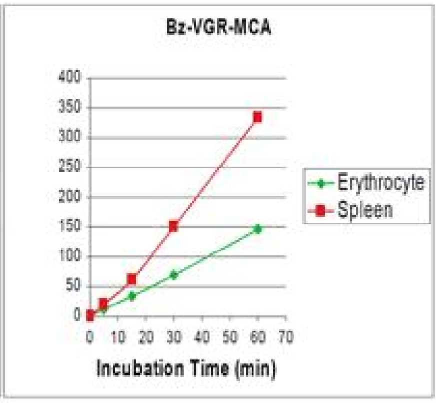

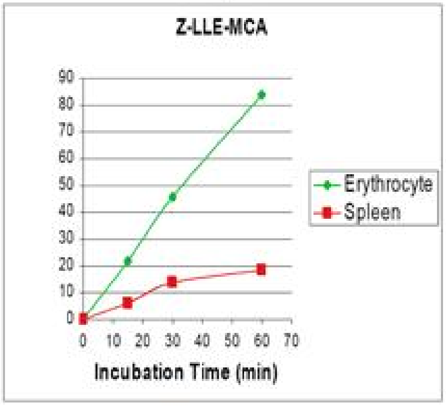

All activities were measured by use of 200µM substrate concentration (final concentration) all dissolved in TEAD buffer. Test system: 20µl enzyme solution (640ng); 20µl substrate solution (incubation at 37°C); 200µl stop solution (100mM sodium chloroacetate dissolved in 30mM Na-acetate, 70mM acetic acid, pH 4.3). Fluorescence was measured in a microplate fluorimeter at 355nm excitation and 460nm emission.

GLOBAL HEADQUARTERS

Enzo Life Sciences, Inc.
10 Executive Blvd
Farmingdale, NY 11735
USA
T 1-800-942-0430
T 1-160-941-0430
F 1-610-941-9252
E info-usa@enzolifesciences.com
www.enzolifesciences.com

EUROPE/ASIA

Enzo Life Sciences (ELS) AG
Industriestrasse 17, Postfach
CH-4415 Lausen
Switzerland
T +41/061 926 89 89
F +41/061 926 89 79
E info-ch@enzolifesciences.com
www.enzolifesciences.com

For Research Use Only, Not for Human

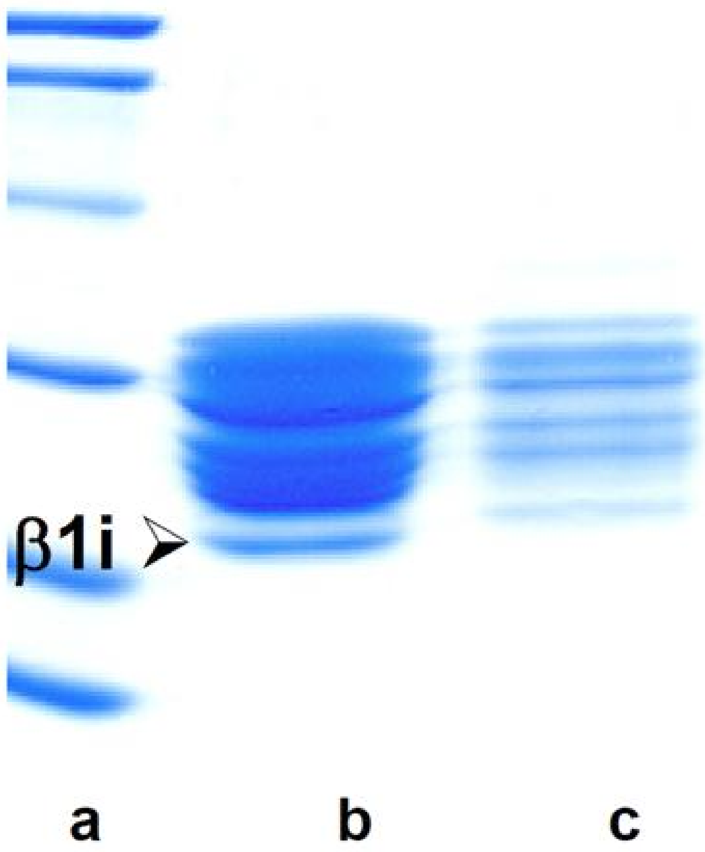

Coomassie stained gel after SDS-PAGE (12.5% acrylamide). Lane (a): molecular weight markers; lane (b) 20S immunoproteosome, spleen-derived (prod.no. BML-PW9645); lane (c): 20S proteosome, erythrocyte-derived (PW8720).

GLOBAL HEADQUARTERS

Enzo Life Sciences, Inc.
10 Executive Blvd
Farmingdale, NY 11735
USA
T 1-800-942-0430
T 1-160-941-0430
F 1-610-941-9252
E info-usa@enzolifesciences.com
www.enzolifesciences.com

EUROPE/ASIA

Enzo Life Sciences (ELS) AG
Industriestrasse 17, Postfach
CH-4415 Lausen
Switzerland
T +41/061 926 89 89
F +41/061 926 89 79
E info-ch@enzolifesciences.com
www.enzolifesciences.com

For Research Use Only, Not for Human

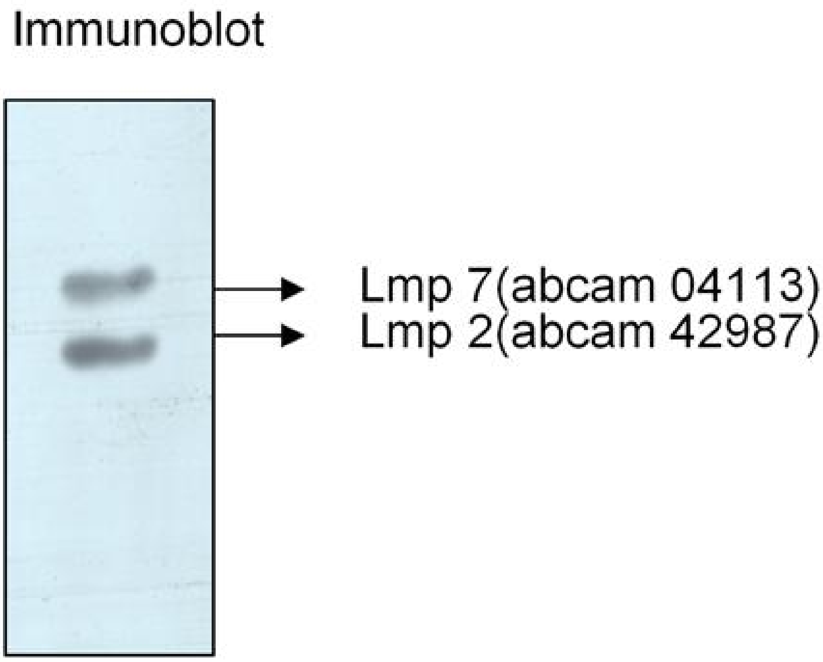

Western blot analysis of Immunoproteasome 20S (human), (purified) (Prod. No. BML-PW9645) probed with anti-Syntaxin 3 antibody (ab4113) and anti-Proteasome 20S LMP2 antibody (ab42987).

Revised 17-Dec-14

GLOBAL HEADQUARTERS

Enzo Life Sciences, Inc.
10 Executive Blvd
Farmingdale, NY 11735
USA
T 1-800-942-0430
T 1-160-941-0430
F 1-610-941-9252
E info-usa@enzolifesciences.com
www.enzolifesciences.com

EUROPE/ASIA

Enzo Life Sciences (ELS) AG
Industriestrasse 17, Postfach
CH-4415 Lausen
Switzerland
T +41/061 926 89 89
F +41/061 926 89 79
E info-ch@enzolifesciences.com
www.enzolifesciences.com

For Research Use Only, Not for Human
