# Supplementary material for: The presence of prolines in the flanking region of an immunodominant HIV‐2 gag epitope influences the quality and quantity of the epitope generated
Source: Eur J Immunol. 2015 Jun 24;45(8):2232–42. doi: 10.1002/eji.201545451 (PMC4832300; doi:10.1002/eji.201545451)
Supplement: Supplementary file 2 — Peer review correspondence [file EJI-45-2232-s002.pdf]

European Journal of Immunology

Peer review correspondence

The presence of prolines in the flanking region of an immunodominant HIV-2 gag epitope influences the quality and quantity of the epitope generated

Sabelle Jallow, Aleksandra Leligdowicz, Holger B. Kramer, Clayton Onyango, Matthew Cotten, Cynthia Wright, Hilton C. Whittle, Andrew McMichael, Tao Dong, Benedikt M. Kessler and Sarah L. Rowland-Jones

Corresponding author: Sabelle Jallow, National Research Foundation, Vaccine Preventable Diseases, Respiratory and Meningeal Pathogens Research Unit, University of the Witwatersrand, South Africa

---

Review Timeline:	Submission date:	5 January 2015
	Editorial decision:	27 January 2015
	Revision received:	2 April 2015
	Second editorial decision:	8 May 2015
	Accepted:	22 May 2015

Handling Executive Committee member: Prof. Annette Oxenius

Please note that the correspondence below does not include the standard editorial instructions regarding preparation and submission of revised manuscripts, only the scientific revisions requested and addressed.

First Editorial Decision – 27 January 2015

Dear Dr. Jallow,

Manuscript ID eji.201545451 entitled "Efficient generation, processing and presentation of an immunodominant HIV-2 gag epitope is strongly influenced by flanking proline residues" which you submitted to the European Journal of Immunology has been reviewed. The comments of the referees are included at the bottom of this letter.

A revised version of your manuscript that takes into account the comments of the referees will be reconsidered for publication.

You should also pay close attention to the editorial comments included below. *In particular, please edit your figure legends to follow Journal standards as outlined in the editorial comments. Failure to do this will result in delays in the re-review process.*

Please note that submitting a revision of your manuscript does not guarantee eventual acceptance, and that your revision will be re-reviewed by the referees before a decision is rendered.

If the revision of the paper is expected to take more than three months, please inform the editorial office. Revisions taking longer than six months may be assessed by new referees to ensure the relevance and timeliness of the data.

Once again, thank you for submitting your manuscript to European Journal of Immunology and we look forward to receiving your revision.

Yours sincerely,
Karen Chu

On behalf of Prof. Annette Oxenius

Dr. Karen Chu
Editorial Office
European Journal of Immunology
e-mail: ejied@wiley.com
www.eji-journal.eu

****

Reviewer: 1

Comments to the Author
The study by Jallow et al. Entitled ‚Efficient generation, processing and presentation of an immunodominant HIV-2 gag epitope is strongyl influenced by flanking proline residues‘ (eji.201545451) investigates the influence of two proline residues flanking an immunodominant HIV-2 gag epitope on CTL generation in infected humans and on epitope processing by 20S constitutive- and immuno-proteasomes in vitro. They find that the presence of these two prolines correlates with stronger CTL responses and with more efficient epitope generation by proteasomes in vitro. Although in vitro cleavage of precursor polypeptides by 20S proteasomes in vitro is rather artificial and although it is uncertain whether such an assay reflects epitope processing from poly-ubiquitylated proteins by 26S proteasomes in cells, the

European Journal of Immunology

correlation is impressive and likely to be informative. Since other in vitro processing assays relying on 26S proteasomes also do not more reliably reflect the complex antigen processing scenario in cells (involving chaperones, deubiquitylating enzymes, adaptors etc) I think it is justified to employ the assay used in this study. It has been shown in several previous studies that residues flanking a CTL epitope affect processing and epitope production as the authors acknowledge in their introduction. However, the present study, which is technicaly solid, shows an impressive correlation of in vitro processing, T cell activation, and viral load in vivo for an important HIV epitope and this is new and should be of major interest.

I have a few suggestions for improvement of the manuscript:

1) In the results section on page 8 it is not explained why the two sets of proline replacements were chosen. This is explained in the Methods section on Peptide design, but I think this section should be moved into the results section on page 8, because the reader will likely pose that question at that point.

2) The constituive proteasome which was used for in vitro digests (purchased from Enzo Life Sciences) was purified from human erythrocytes which is fine as erythrocytes usually do not contain immunoproteasome. The immunoproteasome used, however, (also purchased from Enzo Life Sciences) was purified from human spleen and this contains only immunoproteasome to 60-80 %. The authors should therefore perform graded western blots of the constitutive and immunoproteasome lot they used with antibodies to beta1 and LMP2 (beta1i) as well as beta5 and LMP7 to characterize the enzyme preparations they work with. Enzo Life Sciences do not show data on this on the product sheet. The authors should also write in the results section from what source tissue the proteasome preparations were.

3) The first paragraph ends with a statement which I think is too strong: ‚can be at least partly attributed to superior antigen procssing and…‘. I suggest to change this passage into ‚correlates with superior antigen processing in vitro and….‘

4) Page 13: ..because both the both the C--- Please correct the word duplication

5) Figure 3: Please explain in the legend what the dots in peptide sequences mean – I suppose they indicate the cleavage sites.

6) In the discussion the authors may want to consider the limitations of the in vitro proteasome digestion assay they used as mirrored in the literature

Reviewer: 2

Comments to the Author

Jallow and co-workers demonstrate that several proline residues in the flanking regionof an HLA-B14 restricted HIV-2 specific epitope facilitate antigen processing and mediate strong CD8 responses and (although not directly demonstrated here) viral control. Overall, the study is well done and the results convincing. I have a few comments that may help to strengthen the paper.

1. With the dominant impact of the proline residues, is it possible to redirect a 'weak' Tcell epitope to a 'strong' epitope by introducing prolines in the respective flanking region?
2. The (previously described) restriction by HLA-B14 should be mentioned in the abstract and introduction.
3. Is there viral selection/evolution away from the proline residues in the B14+ subjects?
4. Are the CD8 responses in figure 1a and b still associated with the viral PPP genotype after removal of the 5 B14+ individuals? If yes, how does the PPP motif affect CD8 responses targeting other viral regions?
5. On page 14, the authors argue that the accumulation of prolines may lead to low dimer stability and thus facilitate antigen processing. It should be mentioned more clearly that this may be an aditional mechanism, idependent from the in vitro peptide processing analyzed here.

Reviewer: 3

Comments to the Author
As compared to HIV-1, HIV-2 infection is characterized by reduced viremia and slower disease progression in which HIV-2-specific CTL responses most likely play a critical role. In particular, CTL responses to an 18 amino-acid region of HIV-2 gag p26 protein (peptide 46) are inversely correlated with plasma viral loads. Peptide 46 contains a well-characterized CTL epitope named DA9. The aa sequence of DA9 is flanked by proline residues (so called PPP-motif) that have been associated with viral control. In the present work, Jallow et al. asked whether the prolines flanking DA9 could modulate the quality and quantity of DA9 epitope generation and thus the magnitude and specificity of CTL responses.

To this end, using a cohort of HIV-2 infected individuals, they first show that a higher magnitude of T-cell responses to peptide 46 significantly correlates with the presence of the PPP-motif in gag p26 of the autologous virus. Using a library of truncated peptide they narrow down CTL responses to the DA9 epitope. Finally, using in vitro digestion, of peptides containing the DA9 peptide, with purified proteasomes and immunoproteasomes, they demonstrate that the presence of the prolines flanking DA9 favours the generation of the exact DA9-epitope and of DA9-epitope precursors including peptides with the exact C-terminal cut required to generate D19. They conclude that the strong gag-specific responses in patients infected with viruses harbouring the PPP-motif, might be, at least partially, due to better antigen processing and presentation of the DA9 epitope flanked by two prolines.

The manuscript is very well written and data convincing. However, I believe that one additional experiment is required to really stress the point that the flanking proline residues enhance DA9 presentation to CTL, meaning comparing the recognition of targets cells expressing HIV-2PP; HIV-2SA or HIV1-PQ by DA9-speficic CTL. Indeed, as mentioned by the authors additional steps might limit antigen presentation (after proteasomal processing), such as TAP transport of peptide precursors. The authors mention in the

European Journal of Immunology

manuscript that they already isolated DA9-specific CTL clones, so this experiment should not be technically challenging.

Minor comments:
- more technical information should be provided on the Elispot assays, e.g. what is the bar in the figure, what is the threshold or the criteria for positivity, etc.
- the rational for using the HIV-2SA peptide should be mentioned in the results section (not only in the M&M)
- page-4, line 34 one should read “HIV-1PQ is an equivalent…”

First revision – authors’ response – 2 April 2015

Reviewer: 1
Comments to the Author
The study by Jallow et al. Entitled ‚Efficient generation, processing and presentation of an immunodominant HIV-2 gag epitope is strongyl influenced by flanking proline residues‘ (eji.201545451) investigates the influence of two proline residues flanking an immunodominant HIV-2 gag epitope on CTL generation in infected humans and on epitope processing by 20S constitutive- and immuno-proteasomes in vitro. They find that the presence of these two prolines correlates with stronger CTL responses and with more efficient epitope generation by proteasomes in vitro. Although in vitro cleavage of precursor polypeptides by 20S proteasomes in vitro is rather artificial and although it is uncertain whether such an assay reflects epitope processing from poly-ubiquitylated proteins by 26S proteasomes in cells, the correlation is impressive and likely to be informative. Since other in vitro processing assays relying on 26S proteasomes also do not more reliably reflect the complex antigen processing scenario in cells (involving chaperones, deubiquitylating enzymes, adaptors etc) I think it is justified to employ the assay used in this study. It has been shown in several previous studies that residues flanking a CTL epitope affect processing and epitope production as the authors acknowledge in their introduction. However, the present study, which is technicaly solid, shows an impressive correlation of in vitro processing, T cell activation, and viral load in vivo for an important HIV epitope and this is new and should be of major interest.
I have a few suggestions for improvement of the manuscript:

1) In the results section on page 8 it is not explained why the two sets of proline replacements were chosen. This is explained in the Methods section on Peptide design, but I think this section should be moved into the results section on page 8, because the reader will likely pose that question at that point.

We thank the reviewer for the useful suggestion have and have included the following sentences to the manuscript: Included in Results section page 8 “To generate our peptides, we aligned HIV-2 p26

European Journal of Immunology

sequences from Caio, Guinea Bissau, accession numbers: GQ485448-GQ485516 [27], as well as other HIV-2 p26 sequences from different HIV-2 groups available online. Using these alignments, we designed 2 representative HIV-2 peptides (Table 1), differing by only 2 amino acids at positions 159 and 178. HIV-2PP represents a 34-mer HIV-2 peptide containing prolines at positions 159, and 178; and HIV-2SA represents another 34-mer HIV-2 peptide of the same sequence except it is flanked by serine (S) and alanine (A) at positions 159, and 178 (SA- most common amino acids, apart from prolines, at positions 159 and 178; see materials and methods). HIV-1PQ is an equivalent HIV-1 peptide from the HXB2 (accession no: K03455) sequence."

2) The constituive proteasome which was used for in vitro digests (purchased from Enzo Life Sciences) was purified from human erythrocytes which is fine as erythrocytes usually do not contain immunoproteasome. The immunoproteasome used, however, (also purchased from Enzo Life Sciences) was purified from human spleen and this contains only immunoproteasome to 60-80 %. The authors should therefore perform graded western blots of the constitutive and immunoproteasome lot they used with antibodies to beta1 and LMP2 (beta1i) as well as beta5 and LMP7 to characterize the enzyme preparations they work with. Enzo Life Sciences do not show data on this on the product sheet.

We agree with the reviewer that this detail is useful and have modified the manuscript to reflect it. From our reading of the literature, we agree that Erythrocytes produce proteasomes exclusively, but we also read that the spleen produces the Immunoproteasomes exclusively [1]. The data sheet we received from Enzo life sciences shows that Western blot and Immunoblot were performed on the immunoproteasome isolate, please see attached data sheet from my lot no.

The authors should also write in the results section from what source tissue the proteasome preparations were.

We have also included the source of the proteasome and immunoproteasome in the results section on page 8; with the following sentence "We subjected these peptides to in vitro digestion with highly purified proteasome (isolated from human erythrocytes) and immunoproteasome (isolated from human spleen) and analyzed the proteolysis products by tandem mass spectrometry." And page 19 of materials and methods "Highly purified 20S proteasome (cat no: BML-PW8720-0050), isolated from human erythrocytes and 20S immunoproteasome (BML-PW9645-0050) isolated from human spleen, bought from Biomol International, Enzo Life sciences were used in our experiments. The certificates of analysis included western blots to show the purity of the proteasomes and immunoproteasomes."

3) The first paragraph ends with a statement which I think is too strong: ‚can be at least partly attributed to superior antigen procssing and…‘. I suggest to change this passage into ‚correlates with superior antigen processing in vitro and….‘

European Journal of Immunology

We agree with the reviewer and have modified the sentence (first paragraph of discussion) to “These results indicate that the significant correlation previously found between the presence of prolines at positions 119, 159 and 178 in the HIV-2 capsid and low viral load, as well as the strong gag-specific responses in patients infected with HIV-2 viruses with the PPP capsid both correlate with superior antigen processing in vitro of the DA9 epitope flanked by two proline residues.”

4) Page 13: ..because both the both the C--- Please correct the word duplication :

Corrected

5) Figure 3: Please explain in the legend what the dots in peptide sequences mean – I suppose they indicate the cleavage sites.

Included in the legend and at the bottom of the diagram

6) In the discussion the authors may want to consider the limitations of the in vitro proteasome digestion assay they used as mirrored in the literature

The reviewer is correct, we have included the following sentence in the discussion, 2nd paragraph on page 13. “A limitation to our study is that in vivo, peptides generated by the proteasome or immunoproteasome may be later subjected to further degradation by cytosolic peptidases, and hence it cannot be excluded that the DA9 epitope within the extended peptides or even the actual epitope may be destroyed before it is presented. However, due to the central role the proteasome plays in epitope generation, and the fact that the proteasome has been known to generate correctly cleaved epitopes that are presented [33-35], indicates that in vitro proteasomal digestions can be reliably used [36-38]. “

Reviewer: 2

Comments to the Author

Jallow and co-workers demonstrate that several proline residues in the flanking regionof an HLA-B14 restricted HIV-2 specific epitope facilitate antigen processing and mediate strong CD8 responses and (although not directly demonstrated here) viral control. Overall, the study is well done and the results convincing. I have a few comments that may help to strengthen the paper. 1. With the dominant impact of the proline residues, is it possible to redirect a 'weak' Tcell epitope to a 'strong' epitope by introducing prolines in the respective flanking region?

The reviewer makes an interesting suggestion, but in our view, this is beyond the scope of this paper: the proposed study would require looking at several epitopes, with different flanking amino acids, as well as prolines. The position of the proline may also be important and we do speculate in the discussion that it may be that prolines can make a weak epitope stronger, but we are not providing evidence for that hypothesis in our paper.

I think several studies involving peptides with prolines naturally in the flanking regions can be done to address this possibility.

We believe that this is an interesting suggestion and introducing flaninking prolines to improve processing is a logical extension of this work.

2. The (previously described) restriction by HLA-B14 should be mentioned in the abstract and introduction.

We agree with the reviewer and have included in the introduction, the following sentence: “The HIV-1 epitope was first reported as the dominant HLA B-14 restricted CTL response in a long term non-progressor, infected for over 20 years with HIV-1” in the introduction on page 6” However due to word count restriction, we could not include any more in the abstract.

3. Is there viral selection/evolution away from the proline residues in the B14+ subjects?

The DA9 epitope is within the MHR of gag, which is highly conserved amongst all primate and even some non-primate lentiviruses. This implies that the MHR plays an important role in retroviral structure and replication. This region also contains several immunodominant epitopes that are restricted by various HLA alleles. Combined, these factors may limit escape mutations. This lack of escape mutations for the immunodominant HIV-1 epitope was also previously documented in the first finding of the DA9 epitope, where it was present, without escape mutations in a LTNP infected for 20 years with HIV-1[2, 3] . Since the majority of the sequences from our samples had proline residues, I think that the virus is constrained either structurally and or functionally to maintain these prolines, as they may be crucial in the viral life cycle or confer protection from other nearby or overlapping CTLs. It’s also possible that strong CTL responses may limit viral replication and therefore the emergence of escape variants.

4. Are the CD8 responses in figure 1a and b still associated with the viral PPP genotype after removal of the 5 B14+ individuals?

YES, the association remains even when the 5 HLA-B + individuals were removed.

If yes, how does the PPP motif affect CD8 responses targeting other viral regions?

In terms of antigen processing, this PPP motif will only affect nearby epitopes, where the prolines either flank or are part of the epitope. While there are several epitopes spanning the PPP area of the capsid, the association between high immune responses and low viral load was only significant for the peptide 46 which only contains the full DA9 epitope. While several other factors could be involved in this association, two papers have indicated that the presence of these prolines makes the HIV-2 capsid more sensitive to trim 5 α restriction; indicating increased proteasomal degradation of the PPP capsid by trim5α (host restriction factor) may further limit viral load [4-6].

5. On page 14, the authors argue that the accumulation of prolines may lead to low dimer stability and thus facilitate antigen processing. It should be mentioned more clearly that this may be an aditional mechanism, idependent from the in vitro peptide processing analyzed here.

The reviewer is correct, and this statement in the discussion has been modified in the text to the following: "...The lower binding energy of the PPP capsid suggests lower dimer stability that may also have consequences for antigen processing, and this mechanism may be additive to the direct influence on proteosomal processing that was documented in the current study. "

Reviewer: 3
Comments to the Author
As compared to HIV-1, HIV-2 infection is characterized by reduced viremia and slower disease progression in which HIV-2-specific CTL responses most likely play a critical role. In particular, CTL responses to an 18 amino-acid region of HIV-2 gag p26 protein (peptide 46) are inversely correlated with plasma viral loads. Peptide 46 contains a well-characterized CTL epitope named DA9. The aa sequence of DA9 is flanked by proline residues (so called PPP-motif) that have been associated with viral control. In the present work, Jallow et al. asked whether the prolines flanking DA9 could modulate the quality and quantity of DA9 epitope generation and thus the magnitude and specificity of CTL responses.
To this end, using a cohort of HIV-2 infected individuals, they first show that a higher magnitude of T-cell responses to peptide 46 significantly correlates with the presence of the PPP-motif in gag p26 of the autologous virus. Using a library of truncated peptide they narrow down CTL responses to the DA9 epitope. Finally, using in vitro digestion, of peptides containing the DA9 peptide, with purified proteasomes and immunoproteasomes, they demonstrate that the presence of the prolines flanking DA9 favours the generation of the exact DA9-epitope and of DA9-epitope precursors including peptides with the exact C-terminal cut required to generate D19. They conclude that the strong gag-specific responses in patients infected with viruses harbouring the PPP-motif, might be, at least partially, due to better antigen processing and presentation of the DA9 epitope flanked by two prolines. The manuscript is very well written and data convincing. However, I believe that one additional experiment is required to really stress

the point that the flanking proline residues enhance DA9 presentation to CTL, meaning comparing the recognition of targets cells expressing HIV-2PP; HIV-2SA or HIV1-PQ by DA9-speficic CTL. Indeed, as mentioned by the authors additional steps might limit antigen presentation (after proteasomal processing), such as TAP transport of peptide precursors. The authors mention in the manuscript that they already isolated DA9-specific CTL clones, so this experiment should not be technically challenging.

We agree with the reviewer that this additional experiment would make our data more convincing, however, we have already experienced several challenges in performing this experiment. Unlike HIV-1, HIV-2 constructs are scarce, including recombinant vaccinia with HIV-2 genes. Thus performing these additional experiments will be very challenging, as it will involve generating recombinant HIV-2 vaccinia viruses or complete HIV-2 pseudoviruses with the specific sequences (PP or SA of gagP26) that would replicate with sufficient vigour to allow high multiplicity of infection in CTL target lines, as well has have a suitable reporter to measure infectivity. Whilst we completely agree that the reviewer’s suggested experiment would significantly enhance our paper, we respectfully ask that our revised paper be considered without this additional work, as we think this would be very difficult to achieve successfully.

Minor comments:
- more technical information should be provided on the Elispot assays, e.g. what is the bar in the figure, what is the threshold or the criteria for positivity, etc.
We agree with the reviewer and have included in the materials and methods, and figure legends the following: “Each dot represents the response from one individual; fifty-one samples were assayed once. The average of the spots from the control wells were subtracted from all the sample wells before computing the SFU/Ml. Results were regarded as positive if results were at least 3 times the mean of the quadruplicate negative control wells and over 50 SFU/106 PBMCs. If background wells were more than 30 SFU/106 PBMCs or if both positive control wells (phytohaemagglutinin or FEC stimulation) were negative, the assay was excluded from further analysis. Black horizontal bars represent median values, and significant P values are indicated. IFN□ immune responses were compared using nonparametric Wilcoxon/Mann-Whitney U test.”

- the rational for using the HIV-2SA peptide should be mentioned in the results section (not only in the M&M)
We agree and have included the following to the results “To generate our peptides, we aligned HIV-2 p26 sequences from Caio, Guinea Bissau, accession numbers: GQ485448-GQ485516 [27], as well as other HIV-2 p26 sequences from different HIV-2 groups available online. Using these alignments, we designed two representative HIV-2 peptides (Table 1), differing by only 2 amino acids at positions 159 and 178. HIV-2PP represents a 34-mer HIV-2 peptide containing prolines at positions 159, and 178; and HIV-2SA represents another 34-mer HIV-2 peptide of the same sequence except it is flanked by serine (S) and alanine (A) at positions 159, and 178. (SA- most common amino acids, apart from prolines, at positions

European Journal of Immunology

159 and 178; see materials and methods). HIV-1PQ is an equivalent HIV-1 peptide from the HXB2 (accession no: K03455) sequence."

- page-4, line 34 one should read "HIV-1PQ is an equivalent

We agree and have changed the sentence to "HIV-1PQ is an equivalent peptide from the HXB2 sequence (accession no: K03455)."

Second Editorial Decision – 8 May 2015

Dear Dr. Jallow,

It is a pleasure to provisionally accept your manuscript entitled "Efficient generation, processing and presentation of an immunodominant HIV-2 gag epitope is strongly influenced by flanking proline residues" for publication in the European Journal of Immunology. For final acceptance, please follow the instructions below and return the requested items as soon as possible as we cannot process your manuscript further until all items listed below are dealt with.

Please note that EJI articles are now published online a few days after final acceptance (see Accepted Articles: http://onlinelibrary.wiley.com/journal/10.1002/(ISSN)1521-4141/accepted). The files used for the Accepted Articles are the final files and information supplied by you in Manuscript Central. You should therefore check that all the information (including author names) is correct as changes will NOT be permitted until the proofs stage.

We look forward to hearing from you and thank you for submitting your manuscript to the European Journal of Immunology.

Yours sincerely,
Karen Chu

on behalf of Prof. Annette Oxenius

Dr. Karen Chu
Editorial Office
European Journal of Immunology
e-mail: ejied@wiley.com
www.eji-journal.eu
